# Anxiety disorder following dental care: About 150 cases

**DOI:** 10.1192/j.eurpsy.2023.459

**Published:** 2023-07-19

**Authors:** L. Tbatou, B. Sofiya, L. Fouad

**Affiliations:** Psychiatric emergency, Hospital ArRazi, Salé, Morocco

## Abstract

**Introduction:**

The dental surgeon may be the cause of a psychological trauma - without necessarily knowing it - as well as being confronted with patients who have undergone a trauma as a result of dental care or with patients who have undergone some kind of trauma and that the dental practice exacerbates their anxieties. In this case, the treatment becomes difficult or even inaccessible or a cause of treatment failure.

**Objectives:**

Study the events that can cause trauma and the different approaches to psychological trauma in dental care.

How to explore the traumatic event in a patient and propose its erasure?

Study the events that can cause trauma and the different approaches to psychological trauma in dental care. How to explore the traumatic event in a patient and propose its erasure?

**Methods:**

This is a descriptive and analytical cross-sectional study based on a hetero questionnaire filled in by patients who consulted a dental surgeon.

**Results:**

41% are men, 76% are aged between 30 and 50 years, for the marital status: 43% are single, 38% are married, 72% have an average socioeconomic level, 36% of patients have a personal history of a psychiatric disorder, 21% personal history of a medical disease, 32% have a disorder related to the use of psychoactive substances,

For the reason of consultation: 28%: dental square, 42%: oral malformations, 17%: gingival problem, 8% dental extraction. Medication used: 65% of patients used anti-inflammatory drugs, 77% used antibiotics, 13% used paracetamol. Duration of treatment: 86% one year

41% are men, 76% are aged between 30 and 50 years, for the marital status: 43% are single, 38% are married, 72% have an average socioeconomic level, 36% of patients have a personal history of a psychiatric disorder, 21% personal history of a medical disease, 32% have a disorder related to the use of psychoactive substances, For the reason of consultation: 28%: dental square, 42%: oral malformations, 17%: gingival problem, 8% dental extraction. Medication used: 65% of patients used anti-inflammatory drugs, 77% used antibiotics, 13% used paracetamol. Duration of treatment: 86% one year .

**Conclusions:**

He should be careful, attentive and open to the principles of the psychology of communication in his psychological approach, in order to adapt the level of difficulties of care to the capacity of comprehension, so as to take care of the patient in his globality and avoid any traumatic act. He should not hesitate to send his patient to a psychiatrist if necessary.

**Disclosure of Interest:**

None Declared

